# Neurohormonal Changes in the Gut–Brain Axis and Underlying Neuroendocrine Mechanisms following Bariatric Surgery

**DOI:** 10.3390/ijms23063339

**Published:** 2022-03-19

**Authors:** Eirini Martinou, Irena Stefanova, Evangelia Iosif, Angeliki M. Angelidi

**Affiliations:** 1 Department of Upper Gastrointestinal Surgery, Frimley Health NHS Foundation Trust, Camberley GU16 7UJ, UK; eirini.martinou@nhs.net; 2 Faculty of Health and Medical Sciences, University of Surrey, Guildford GU2 7XH, UK; 3 Department of General Surgery, Frimley Health NHS Foundation Trust, Camberley GU16 7UJ, UK; irena.stefanova@nhs.net; 4 Department of General Surgery, Royal Surrey County Hospital, Guildford GU2 7XX, UK; e.iosif@nhs.net; 5 Division of Endocrinology, Boston Children’s Hospital, Harvard Medical School, Boston, MA 02115, USA; 6 Broad Institute of MIT and Harvard, Cambridge, MA 02142, USA

**Keywords:** bariatric surgery, gut–brain axis, neuropeptides, central nervous system, gut peptides, appetite, energy homeostasis, gut microbiota

## Abstract

Obesity is a complex, multifactorial disease that is a major public health issue worldwide. Currently approved anti-obesity medications and lifestyle interventions lack the efficacy and durability needed to combat obesity, especially in individuals with more severe forms or coexisting metabolic disorders, such as poorly controlled type 2 diabetes. Bariatric surgery is considered an effective therapeutic modality with sustained weight loss and metabolic benefits. Numerous genetic and environmental factors have been associated with the pathogenesis of obesity, while cumulative evidence has highlighted the gut–brain axis as a complex bidirectional communication axis that plays a crucial role in energy homeostasis. This has led to increased research on the roles of neuroendocrine signaling pathways and various gastrointestinal peptides as key mediators of the beneficial effects following weight-loss surgery. The accumulate evidence suggests that the development of gut-peptide-based agents can mimic the effects of bariatric surgery and thus is a highly promising treatment strategy that could be explored in future research. This article aims to elucidate the potential underlying neuroendocrine mechanisms of the gut–brain axis and comprehensively review the observed changes of gut hormones associated with bariatric surgery. Moreover, the emerging role of post-bariatric gut microbiota modulation is briefly discussed.

## 1. Introduction

Obesity is a global public health issue that has been increasing in frequency at an alarming rate in recent decades [1]. According to World Health Organization statistics, the worldwide prevalence of obesity almost tripled between 1975 and 2016 [2,3,4]. It is estimated that 2 billion adults are overweight and of those, 650 million have obesity with a Body Mass Index (BMI) of ≥30. In particular, 39% of adults (≥18 years old) are overweight, and 13% of those have obesity. Linear time trend forecasts suggest that almost 51% of the population will have obesity by 2030 [5]. Obesity is recognized as a leading public health hazard as it is linked with the occurrence of life-threatening diseases and a reduction in life expectancy by 5–20 years [2,6,7].

**Figure 1:**
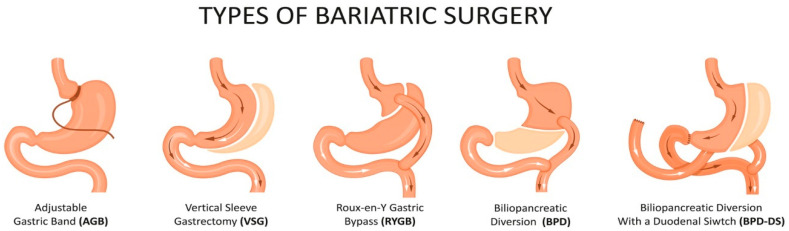
Types of commonly performed bariatric surgery procedures. (Source: https://www.istockphoto.com/, accessed on 14 August 2021).

The cornerstone for the management of obesity is an alteration in lifestyle with dietary changes and an increase in physical activity [8]. However, in some cases, lifestyle interventions and pharmacotherapy may not be sufficient to achieve weight loss goals and maintain long-term weight loss effects, especially in individuals with severe obesity or those overweight with related comorbidities. For individuals in these categories, bariatric surgery (BS) is considered to be safe, and it is an effective alternative treatment [8,9]. Currently, the most commonly performed surgical modalities are vertical sleeve gastrectomy (VSG), gastric bypasses, including the Roux-en-Y gastric bypass (RYGB) and mini(one-anastomosis) gastric bypass and biliopancreatic diversion with duodenal switch (BPD-DS), as shown in Figure 1 [10]. Procedures such as the jejunoileal bypass, vertical banded gastroplasty and adjustable gastric banding (AGB) have become obsolete due to their poor efficacy, adverse effects and high re-operation rates [10].

BS appears to be a safe and successful way to manage obesity, and therefore, the guidelines recommend this approach for patients with a BMI of ≥40, or ≥35 for those with obesity-related comorbidities [8,11]. Long-term observational studies have reported not only sustainable weight loss but also improvements in the metabolic profile after BS, especially in terms of glycemic and lipid metabolism [12]. There is strong evidence suggesting that compared to lifestyle intervention and pharmacotherapy, BS may result in superior glycemic control, often leading to the remission of diabetes, while reducing cardiovascular risk factors in patients with type 2 diabetes mellitus (T2DM) [13,14]. Studies including the SOS, STAMPEDE and CROSSROAD trials have demonstrated the effectiveness of BS in T2DM remission and prevention [15,16,17]. Specifically, Cummings et al. reported a 60% T2DM remission after RYGB vs. 6% remission after medical therapy [15]. Additionally, Carlsson et al. showed that BS may reduce the risk of T2DM development by 96%, 84% and 78% after 2, 10 and 15 years, respectively [16]. Therefore, the current guidelines recommend BS for adults with T2DM and a BMI of ≥40 or those with a BMI of ≥35 who have inadequate glycemic control and cannot maintain weight loss with non-surgical treatments. Moreover, bariatric surgery should also be considered as a treatment option for adults with T2DM and a BMI of 30.0–34.9 who have inadequate glycemic control and cannot achieve sustainable weight loss despite lifestyle changes and optimal treatment. [13,14,18]. Additionally, BS is associated with both short- and long-term improvement in dyslipidemia, as shown by the STAMPEDE trial, which reported reductions in triglyceride levels of 40% and 29% in the RYGB and VSG groups, respectively. In comparison, a reduction of 8% was reported for the medical therapy group [10,19]. BS has been associated with hypertension remission in 43% to 83% of patients within one year of the procedure [10]. Remission from obstructive sleep apnea, osteoarthritis and urinary incontinence as well as reductions in the risk of developing obesity-related cancers such as colon, liver and breast have also been reported after BS [10].

Obesity is significantly associated with an elevated risk for developing cardiovascular disease and cancer, thereby having a negative impact on quality of life and increasing the risk of all-cause mortality [6,20]. Several studies have suggested that BS is associated with lower all-cause mortality rates and greater life expectancy than usual methods of obesity care because it decreases the risk developing diseases and related comorbidities in the future [21,22,23].

BS usually leads to a body weight reduction of 25% to 35% within 1–2 years, primarily through the restriction of food intake and/or malabsorption [24]. Various mechanisms have been proposed to explain the weight loss achieved through BS, and the restriction of food intake due to the reduction in the size of the gastric pouch is an important contributor to this, as it results in a reduction in caloric intake [25]. However, the exact physiological changes and mechanisms underlying the post-operative decrease in caloric intake and body weight are poorly understood [25]. Decreased appetite and early satiety are likely to be not only due to the decrease in gastric space but may be contributed to by neuroendocrine modulation after BS [26]. Complex interactions between the brain and hormone productions in the gastrointestinal (GI) tract, pancreas, liver and adipose tissue are considered to contribute substantially to the effects of BS [25]. Gut peptides that cross the blood–brain barrier and cause changes in neural activation are likely to play roles in the benefits of BS [25]. However, the exact physiological changes and mechanisms underlying the post-operative decrease in caloric intake and body weight have not been fully elucidated [25]. This review aims to comprehensively describe the neuroendocrine changes that occur in the gut–brain axis following BS along with their underlying mechanisms. Therefore, a systematic literature review, following the Preferred Reporting Items for Systematic reviews and Meta-Analyses (PRISMA) guidelines, was not performed. We searched for original articles and relevant reviews in PubMed, Google Scholar and Web of Science databases using terms related to the sections discussed in the current review without applying restrictions in publication time. Articles published in a language other than English were excluded. The present review summarizes the potential neuroendocrine mechanisms of the gut–brain axis and highlights the observed alterations in gut hormones after bariatric surgical procedures. Finally, the related post-bariatric gut microbiota modulation is briefly discussed.

## 2. The Role of the Nervous System in Appetite and Energy Regulation

### 2.1. Central Nervous System Related Mechanisms

Research has shown that the central nervous system (CNS) plays a fundamental role in modulating appetite, satiety and energy balance by acting through both the brain and the peripheral organs [27]. Most evidence in this area comes from experimental studies on rodents, where increased obesity has been observed in animals with hypothalamic lesions or with functional disruption of the hypothalamus [28,29]. CNS regulation of appetite and body weight in the human brain is a complex process controlled by several neural systems that integrate myriad cognitive, emotional, hedonic and homeostatic pathways involved in energy expenditure and obesity [27,30]. The primary homeostatic regulatory area in the CNS is the hypothalamus, which consists of distinct nuclei including the arcuate nucleus (ARC), the paraventricular nucleus (PVN), the lateral hypothalamic area (LHA), the dorsomedial nucleus (DMN) and the ventromedial nucleus (VMN) [30].

The ARC is located adjacent to the median eminence and is one of the most well-characterized brain regions related to the control of appetite. It contains two distinct neuronal populations with opposing effects: orexigenic agouti-related peptide (*AgRP*)/neuropeptide Y (*NPY*) neurons and anorexigenic pro-opiomelanocortin (*POMC*) neurons [30,31]. Orexigenic neuropeptides *AgRP*/*NPY* are co-expressed and have been found to be elevated under fasting and decreased under feeding conditions. *NPY* is expressed throughout the brain; however, it is densely expressed in the ARC. It acts through its five receptors (Y1, Y2, Y3, Y4 and Y5), with Y1 and Y5 being responsible for mediating the effects of *NPY* on energy balance [32]. Yang et al. showed that the overexpression of *NPY* increased food intake in rats, whereas knockdown with RNA interference was correlated with a 10% reduction in food intake [33]. The second orexigenic neuropeptide *AgRP* is exclusively expressed within the ARC. Experimental studies have shown that its overexpression triggers food intake and reduces energy expenditure, leading to obesity [34,35]. In addition, *AgRP*/*NPY* neurons produce γ-amino-butyric acid (GABA), by which they exert their orexigenic effects through the inhibition of anorexigenic *POMC* neurons [27,36]. Experimental studies have shown that a loss of GABAergic signals not only in the ARC but also in other areas such as the LHA promotes anorexia in mice [37,38].

On the other hand, anorexigenic *POMC* neurons express *POMC*, which is cleaved post-translationally, generating several bioactive peptides, with the most important being the α-melanocyte-stimulating hormone (*α-MSH*) [32]. Expression levels of *POMC* and *α-MSH* have been observed to be elevated in the fed condition and decreased in the fast condition, resulting in the suppression of appetite and food intake [39]. Experimental gene modulation studies in mice have shown that the overexpression of *α-MSH* results in reductions in weight gain and adiposity as well as an improvement in glucose tolerance [40]. *α-MSH* is an agonist of the melanocortin 3 (*MC3R*) and 4 (*MC4R*) receptors, which have been found to control appetite [41]. *MC3R* and *MC4R* knockout mice develop obesity while control mice do not, but the obesity phenotype differs between the two knockout variants [42]. *MC3R* knockout mice demonstrate a mild obese phenotype with increases in body weight and body fat [43]. On the contrary, *MC4R* knockout mice demonstrate hyperphagia and develop severe obesity and T2DM [44]. The mechanism through which *α-MSH* acts on either the *MC3R* or *MC4R* receptors is not completely understood, and further research is required to elucidate how this neuropeptide regulates food intake and energy balance [32]. Cocaine and amphetamine-regulated transcript (*CART*) is another anorexigenic neuropeptide that is abundant in the ARC and is co-expressed with *POMC* [45]. Although the mechanisms by which *CART* regulates food intake are poorly understood, studies have shown that *CART* infusion inhibits food intake and stimulates the thermogenesis of brown adipose tissue (BAT) [46].

The PVN is an important area within the hypothalamus that plays a significant role in the regulation of energy homeostasis [30]. The PVN is located to either side of the third ventricle roof and receives neuronal projections from the ARC *AgRP*/*NPY* and *POMC* neurons as well as from extrahypothalamic regions [47]. The PVN demonstrates the highest level of *MC4R* expression within the CNS, and the disruption of *MC4R* in the PVN results in hyperphagia and reduced energy expenditure, leading to the development of the obesity phenotype [48]. In addition to the PVN, *AgRP*/*NPY* and *POMC* neurons of the ARC are also projected to the VMN which, in turn, projects to other hypothalamic areas and the brainstem [30]. The VMN has an abundant population of glycoresponsive neurons, and brain-derived neurotrophic factor (*BDNF*) is highly expressed [49]. *BDNF* acts through its receptor Tropomyosin receptor kinase B (*TRKB*) to regulate appetite in humans and mice [49]. Experimental studies have shown that a *TRKB* mutation results in hyperphagia and weight gain, whereas central *BDNF* administration is associated with decreased food intake [50]. In addition, the PVN and VMN contain endocannabinoid receptors 1 and 2 (*CB1R* and *CB2R*), which have been demonstrated to have significant effects on metabolism and appetite. The activation of the endocannabinoid system potentially contributes to hyperphagia, decreased energy expenditure, obesity and metabolic syndrome through the involvement of appetite modulators such as melanin-concentrating hormone (MCH), leptin and glucocorticoids within the hypothalamus [51,52]. Cardinal et al. showed an association between hypothalamic *CB1R* knockout in mice and an increased energy balance as well as decreased weight gain in comparison with a wild-type group while receiving a normocaloric diet [53]. In addition, the administration of leptin caused hypophagia in the wild-type group but not in the *CB1R*-knockout mice [53].

The DMN serves as an important hypothalamic area that is involved in the regulation of appetite and other physiological processes (thermoregulation and stress) [30]. The DMN receives *NPY* and *α-MSH* neurons from the ARC with *α-MSH* demonstrating inhibitory actions against *NPY* [51]. The DMN has been found to have cholecystokinin (CCK) receptors, and in experimental studies involving *CCK-1* knockout rats, hyperphagia and the upregulation of *NPY* gene expression were observed in the DMN [54]. Additionally, de La Serre et al. showed that the in vivo overexpression of the *NPY* gene in DMH reduces CCK-induced satiety, suggesting that *CCK-NPY* signaling may be a potential mechanism by which the DMN regulates energy homeostasis [55].

The LHA is another hypothalamic area. It is rich in orexin and MCH neurons and receives downstream projections from the ARC [51]. Studies have shown that the presence of LHA lesions reduces body weight and adipose tissue in rats, indicating that it may play a critical role in regulating appetite [56,57]. Orexin A and B are produced from orexin neurons in the LHA and are important neuropeptides that are involved in the regulation of energy balance [58]. Orexin A acts through its receptors, orexin 1 receptor (OX1R) and orexin 2 receptor (OX2R), to increase food intake as well as induce behavioral changes related to food reward [58,59]. Similar orexigenic activity is displayed by MCH, as demonstrated by experimental studies which showed that MCH gene overexpression resulted in increased food intake and obesity in mice [60]. Conversely, MCH-knockout mice demonstrated increased energy expenditure and were resistant to diet-induced obesity [61].

In addition to the hypothalamus, other extrahypothalamic areas contribute to appetite and energy balance regulation. The midbrain influences hedonic feeding behavior through dopaminergic neuronal pathways and is considered to be the root cause of obesity [31]. The amygdala, hippocampus and prefrontal cortex are cognitive decision-making centers that produce signals that integrate with hypothalamic signals to control homeostasis regulation [47].

**Figure 2:**
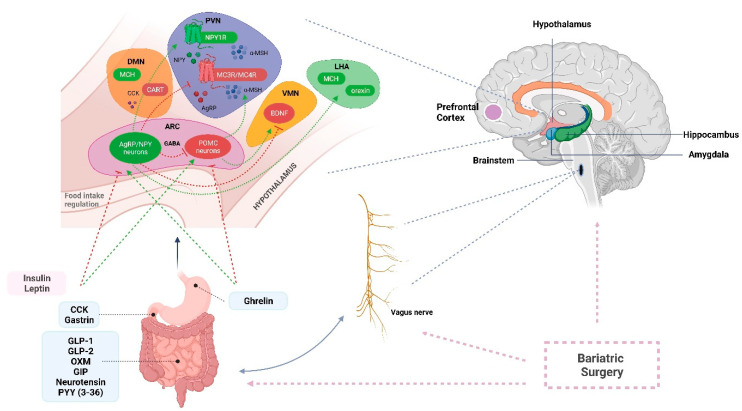
Neurohormonal circuits and neuronal signals derived from the hypothalamus and other centers regulating appetite and energy homeostasis, which may be affected by bariatric surgery. (Created in https://biorender.com/, accessed on 13 February 2022) *AgRP*: agouti-related peptide, ARC: arcuate nucleus, *BDNF*: brain-derived neurotrophic factor, *CART*: cocaine and amphetamine-regulated transcript, CCK: cholecystokinin, DMN: dorsomedial nucleus, GABA: γ-amino-butyric acid, GIP: gastric inhibitory polypeptide, GLP: glucagon-like peptide, LHA: lateral hypothalamic area, MCH: melanin-concentrating hormone, *MC3R*: melanocortin 3 receptor, *MC4R*: melanocortin-4 receptor, *NPY*: neuropeptide Y, OXM: oxyntomodulin, *POMC*: pro-opiomelanocortin, PYY: peptide YY, PVN: paraventricular nucleus, VMN: ventromedial nucleus.

A summary of the CNS signals involved in the regulation of appetite and satiety is shown in Figure 2.

### 2.2. Autonomous-Nervous-System-Related Mechanisms

The autonomic nervous system (ANS) includes the sympathetic (SNS) and parasympathetic nervous system (PNS) and plays a key role in the regulation of body weight through communication between the CNS and the GI system [62]. The SNS appears to be important for processes related to energy expenditure as well as the storage of fat. Its activation results in the mobilization of white adipose tissue (WAT), which is the main form of energy storage [62,63]. Experimental studies in rats and humans have found that increased SNS activity results in an increased lipolysis rate in adipose tissue, whereas sympathetic denervation inhibits lipid mobilization and increases the fat pad mass [64,65,66,67]. Bartness et al. showed the presence of important neuronal connections between the CNS, SNS and WAT and hypothesized that the lipid accumulation which is observed in obesity could be attributed to a reduced SNS activity rather than increased parasympathetic activity [64,68,69]. In addition to its role in lipolysis, the SNS is crucial for energy expenditure as it regulates brown adipose tissue (BAT) thermogenesis [64]. The presence of BAT has been negatively correlated with the body fat content, and when recruited, can contribute to a reduction in body fat [70]. Sympathetic nerve terminals have high *CB1R* expression, indicating that endocannabinoid signaling can affect the regulation of energy metabolism by the SNS [71]. Studies have shown that cannabinoid signaling downregulates the production of proteins involved in thermogenesis, such as uncoupling protein-1 (*UCP-1*), and therefore leads to BAT inhibition [71,72,73,74].

The PNS, through the vagus nerve, appears to represent an important link between the brain and gut. The vagus nerve is a critical modulator of appetite and food intake and a crucial component of the gut–brain axis, as it conveys signals containing information about peripheral gut hormones, nutritional content and visceral distention to the hypothalamus and other brain areas [75]. Its dorsal motor nucleus is located within the brainstem, and with the nucleus of tractus solitarius (NTS), it forms the dorsal vagal complex (DVC) in the brainstem [75]. Vagal branches carry afferent satiety signals from the stomach and other gut regions to the brainstem determining satiety and regulating hunger [76]. The importance of the vagus nerve in controlling food intake and body weight has been demonstrated by several experimental studies which reported that vagal stimulation leads to a reduction in food intake, body weight gain and adipose tissue accumulation through increased CNS satiety signals, whilst a vagotomy has been shown to result in hyperphagia in laboratory animals [77,78,79].

Lastly, one of the main divisions of the ANS is the enteric nervous system (ENS), which is derived from neural crest cells and governs the motility of the GI tract [80]. The ENS is found in two forms: the myenteric plexus located between the longitudinal and circular muscle layers in the GI tract and the submucosal plexus located in the submucosa layer [80]. The ENS is characterized as the “second brain”, as it can operate independently from the SNS and PNS through neurotransmitters similar to those that used by the CNS (serotonin, GABA, *NPY* and endocannabinoids) that are involved in appetite regulation and energy metabolism [81,82]. The ENS is significantly influenced by changes in gut microbiota that lead to alterations in appetite-regulatory neurotransmitters. These alterations may affect aspects of gut motility related to gastric emptying and intestinal transit [80]. Bravo et al. observed that modifications to the animal gut microbiome after treatment with *Lactobacillus rhamnosis* led to changes in the mRNA expression of GABA in CNS regions [83]. Additionally, Bercik et al. found that *BDNF* expression in the hippocampus was altered after the use of antibiotics [84]. There is emerging evidence that the gut microbiota could participate in the gut–brain axis via the ENS; however, the exact mechanism involved and how this affects eating behavior and regulates appetite remains unknown, indicating the need for further research [85].

## 3. Effects of Bariatric Surgery on Nervous System

### 3.1. Central Nervous System and Neuropeptide Changes after Bariatric Surgery

There is experimental evidence that bariatric surgery can induce changes in neuropeptide expression in the hypothalamus and therefore alter food intake and energy balance. However, the data reported by experimental studies with regard to neuropeptide expression changes in the ARC are conflicting. For instance, Romanova et al. investigated changes in neuropeptide expression in hypothalamic areas after RYGB in rats. This study showed that animals that were subjected to RYGB demonstrated a reduction in body weight in comparison with controls and observed a 43% decrease in *NPY* expression and a 35% increase in *α-MSH* mRNA expression in the ARC [86]. On the other hand, a study by Barkholt et al. demonstrated the upregulation of *AgRP*/*NPY* in the ARC with no changes in *CART* and *POMC* mRNA levels post RYGB [87]. Further studies are needed to elucidate the alterations induced by BS in terms of hypothalamic neuropeptide expression. BS also appears to affect the PVN of the hypothalamus through the *MC4R* pathway [88,89]. The hypothesis that *MC4R* signaling plays an important role in the effect of BS on weight loss is supported by in vivo studies showing that mice deficient in *MC4R* lost significantly less weight after RYGB in comparison with mice that had a functional copy of the *MC4R* gene [88]. Moreover, human studies showed that carriers of an *MC4R* variant (I251L) appeared to show greater weight loss after RYGB compared with the non-carriers [89]. A potential mechanism by which RYGB controls food intake is through a positive feedback loop where the enteroendocrine *MC4R* produced by the L-cells upregulates the expression of gut-related peptides (hormone peptide YY (PYY), glucagon-like peptide-1 (GLP1), resulting in the increased production of *MC4R* in the CNS [90].

An alteration in *BDNF* expression was suggested as a potential neuroendocrine outcome after bariatric surgery by Muñoz-Rodríguez et al., who showed that serum *BDNF* was significantly downregulated after RYGB in patients with problematic eating behaviors who had experienced weight regain [91]. A common *BDNF* polymorphism (rs6265, C > T) in which valine is replaced by methionine at codon 66 (Val66Met) has been associated with a lower BMI [92]. A recent longitudinal study by Pena et al. examined the effects of rs6265 polymorphism on weight loss after BS and reported that patients carrying the Met allele demonstrated a loss of 19% more weight than those with the Val allele in the absence of T2DM (*p* < 0.05) [93]. Interestingly, a recent multicenter, longitudinal, observational study by Ciudin et al. proposed a clinical–genetic risk score that could be used as a predictive model for weight loss after BS [94]. This model consists of clinical variables and nine single-nucleotide polymorphisms and has been demonstrated to have a significantly better diagnostic ability with an area under the curve of 0.845 (95% CI: 0.800 to 0.888) compared to the use of clinical variables only (*p* = 0.018) [94]. Amongst the SNPs, *MC4R* and *BDNF* neuropeptides are included in the model, highlighting the association of BS with the CNS in the weight loss process [94].

BS may affect the DVN via alterations in the CCK levels. Although in vivo rat studies have shown no changes in the CCK levels after BS, human studies have reported an increase in the CCK levels post-prandially after RYGB [95,96]. Interestingly, Mumprhey et al. showed the hypertrophy of the CCK-expressing enteroendocrine cells (EECs) located in the Roux limb after RYGB in rats [97]. As a neuropeptide, CCK can activate its receptors and transmit satiety signals to the DMN. An increased CCK level after RYGB could potentially result in greater *NPY* suppression and increase forward feedback, leading to reduced food intake [25].

Additionally, neuropeptide alterations have been observed in the LHA after BS. Studies have shown that orexin levels are affected by bariatric surgery [58]. Gupta et al. noted a decrease in orexin levels in some patients, whereas others had an increase in this neuropeptide after BPD-DS in the early post-operative period and before any weight loss [98]. Interestingly, patients with increased orexin levels demonstrated better glucose and lipid profiles [98]. On the contrary, a study by Cigdem et al. observed significant weight loss and decreased orexin levels after laparoscopic GB [99]. The exact mechanism by which BS affects the orexin levels and how this contributes to the effects seen after BS is still unclear and most likely involves complex neuro-hormonal feedback loops [58]. Barkholt et al. explored the effect of RYGB on MCH in the LHA and ARC in rats [87]. Although the authors observed the significant upregulation of the ARC orexigenic signals in terms of the overexpression of *AgRP* and *NPY* in RYGB rats compared with sham controls, there was no effect on downstream signals in terms of MCH expression in the LHA [87]. The authors suggested that RYGB may stop the orexigenic signals arising from the ARC and eventually do not translate into hunger and food-seeking behaviors [87,100].

There is growing literature evidence that the hypothalamus is affected by BS, and this has been suggested to potentially explain the beneficial effects of bariatric surgery on weight loss and metabolism [101]. Observational neuroimaging studies conducted in humans using functional magnetic resonance imaging (fMRI) demonstrated that bariatric surgery can induce changes in the hypothalamus [102]. In a case–control, cross-sectional study, Frank et al. recorded lower hypothalamic activity during fMRI following the presentation of high-calorie foods for women who had undergone RYGB in comparison with women with obesity. Additionally, women who had undergone RYGB showed similar hypothalamic activity to normal-weight women, suggesting the potential normalization of the CNS after BS [102,103]. Similarly, van de Sandee-Lee et al. reported a change in hypothalamic activity during fMRI in patients with obesity who had undergone RYGB, demonstrating similar activity to lean individuals and suggesting a normalization of obesity-induced hypothalamic dysfunction after BS [104].

Neuroimaging studies have shown that BS affects brain areas other than the hypothalamus. Zeighami et al. investigated the changes in regional brain activity with fMRI in patients with obesity undergoing BS (RYGB, BPD-DS and VSG) [105]. The authors reported an increase in brain activity after surgery in the prefrontal and temporal cortexes, the temporal gyrus and the precuneus, as well as an increase in gray matter [105]. Additionally, the authors observed that the increase in neural activity was significantly associated with weight loss and a better metabolic profile in terms of diastolic blood pressure and waist circumference [105]. The brain reward system was also shown to be affected by BS in neuroimaging studies by Ochner and colleagues, who observed less activation of the mesolimbic reward system after RYGB, as well as a reduction in the activation of brain areas involved in the desire for food [90,106,107].

### 3.2. Autonomous Nervous System Changes after Bariatric Surgery

BS, especially RYGB, has been hypothesized to potentially reduce SNS activity while increasing splanchnic selective sympathetic nerve activity, activating thermogenesis through BAT and increasing energy expenditure; however, findings from previous studies are contradictory [108]. Hankir et al. used 18F-FDG PET-CT imaging to measure BAT 18F-FDG uptake in control and RYGB animals and reported no difference in BAT thermogenesis between groups as well as no difference in *UCP-1* mRNA expression [109]. On the other hand, Baraboi et al. found that a VSG animal group demonstrated increased BAT uptake in comparison with control groups, indicating that VSG may enhance BAT thermogenesis [110]. Curry et al. used microneurography to record sympathetic activity and observed that patients who underwent RYGB had decreased sympathetic muscular nerve activity compared to individuals with obesity; however, no alterations in resting energy expenditure were identified [111]. Interestingly, a recent animal experimental study showed that exposure to cold temperature resulted in a significant increase in BAT sympathetic activity in RYGB rats in comparison with controls as well as the significant upregulation of the *UCP-1* protein [112]. Thermoneutrality reversed the RYGB-induced increase in sympathetic activity, indicating the plasticity of the SNS, which may be affected by BS. The authors of this study concluded that the post-RYGB BAT plasticity could contribute to the metabolic effects that follow BS [112]. Although the exact mechanism by which BS affects the SNS and its potential impact on energy expenditure is still not known, endocannabinoid signaling seems to play an important role in this process. Ye et al. showed that BS, specifically RYGB, increases splanchnic sympathetic activity, inducing BAT thermogenesis and augmenting a resting metabolic rate in rats [113]. Interestingly, treatment of the obesity-control animal groups with a CB1 antagonist appeared to mimic the effects of RYGB on energy balance and weight, whereas treatment of the RYGB animal group with a CB1 agonist resulted in greater weight gain in the RYGB-treated group compared with the RYGB-control group. The authors concluded that CB1 plays a pivotal role in the regulation of energy balance through pathways in which the SNS is involved [113].

BS, especially RYGB, alters the GI anatomy by reducing the gastric volume. In this respect, Bjorklund et al. reported that the meal size post-RYGB appears to be predicted by the pressure recorded in the Roux limb, suggesting that the rapid distention of the Roux limb from food entry is detected by the vagal mechanoreceptors, creating a signal through the CNS to reduce food intake [76,114]. As a result of unintentional peri-operative damage to the vagal branches, BS may lead to the remodeling of gut–brain communication [115]. Ballsmider et al. used fluorescent staining to show that RYGB resulted in a decrease in vagal afferents in the NTS and microglia activation within the vagal structures, whereas after VSG, an increase in the NTS vagal afferents was observed, indicating the possible neuronal re-organization of the hindbrain feeding centers [114,115]. Additionally, the vagus nerve plays an important role in gut–brain communication due to its inherent plasticity, whereby it can change its sensitivity in response to hormonal changes related to nutritional status [75]. Several hormonal receptors, including the CCK-receptor, the GLP-1 receptor and the leptin receptor, are present in the vagal afferents and can transfer satiety signals to the CNS regulating food intake [75]. These neural connections of the vagus nerve in the gut–brain axis may play an important role in weight loss post-BS [116]. Interestingly, despite the plethora of studies that exist with regard to the effect of vagotomy on weight loss during BS, the results remain controversial [116].

It is evident that the nervous system plays a crucial role in the regulation of appetite, food intake and energy balance and that bariatric surgery may cause the re-organization of driving neural signals responsible for appetite and energy control [117]. Alterations in neurohormonal gut peptides, as well as microbiota products, play significant roles in the effects of bariatric surgery through the gut–brain axis and are discussed in the next section.

## 4. Enteroendocrine Effects of Bariatric Surgery on the Gut–Brain Axis

The gut–brain axis is a complex, bidirectional communication network that involves signaling between the gastrointestinal system and the brain. This network is regulated at the neural, hormonal and immunological levels, and its main components are the CNS, the neuroendocrine and neuroimmune systems, the ANS, the ENS and the gut microbiota [108,118,119,120]. At the gut level, the EECs, which lie in the intestinal epithelium, play a major role in gut–brain signaling through the secretion of multiple gut hormones in response to pre-absorptive nutrients [121]. There are various subtypes of EECs that differ in terms of their locations in the GI tract and the gut peptides they secrete. For instance, gastric P/D1 cells produce ghrelin, whilst gastric chief cells produce leptin; I-cells and K-cells in the proximal small intestine secrete CCK and glucose-dependent insulinotropic (GIP) hormone, respectively; L cells in the distal small bowel produce GLP-1, GLP-2, oxyntomodulin (OXM) and PYY [122].

Gut hormones are involved in endocrine signaling with entry to the systemic circulation and affect peripheral targets such as the brain. They also affect paracrine signaling with the activation of vagal and spinal afferents which transmit signals to the brain [122]. Vagal afferent fibers extend to the lamina propria of the intestinal villi, terminating very close to the basolateral surface of the EECs, where they express receptors for ghrelin, leptin, GLP-1 and PYY. The activation of these receptors leads to neuronal firing [123]. Furthermore, the ENS is also involved in the gut–brain neuronal signaling axis, as ENS neurons are located close to EECs, and the afferent fibers also express gut hormone receptors. This pathway of communication to the brain is indirect, as it involves the initial activation of the vagal and spinal afferents in the gut [122,124,125].

The gut–brain axis plays a crucial role in maintaining energy homeostasis. GI hormones are key mediators that directly influence hunger and satiety and interact with the CNS to control energy balance [126]. The alteration of gut peptide levels and neuroendocrine mechanisms following weight-loss surgery is linked to changes in eating behavior, post-operative weight loss and a reduction in metabolic disease.

### 4.1. Ghrelin

Ghrelin is a 28-amino-acid peptide (Orexigen) produced mainly by the P/D1 cells in the gastric fundus with some expression in the ARC of the hypothalamus, pituitary gland, pancreas, adrenal gland, lungs, skeletal muscles, testes, ovaries and small bowel [127,128]. Ghrelin plays a vital role the regulation of food intake, body weight and glucose homeostasis. It is also described as the main “hunger hormone”. The plasma level of ghrelin rises in a fasted state and falls within the first hour after a meal in proportion to the calories consumed and types of macronutrients, with a greater decrease with carbohydrate-rich meal compared to a high-fat meal [129,130]. Individuals with obesity have lower ghrelin levels and considerably reduced post-prandial suppression in comparison to normal-weight individuals [131].

Due to its dual expression, ghrelin exhibits its effects by acting both peripherally and centrally. The central administration of ghrelin leads to direct orexigenic action on the hypothalamus, whilst peripherally, ghrelin induces the secretion of growth hormone factor and activates the gut–brain axis to increase food intake [132]. The effects on appetite are mediated by the *AgRP*/*NPY* peptides acting on the ARC in the hypothalamus [133].

There is controversial data on the effect of BS procedures on the ghrelin concentration [133]. Following an AGB, the majority of studies have shown an increase in the ghrelin concentration post-operatively within 6 to 24 months [134,135,136,137,138,139]. However, some authors have reported a decrease in ghrelin, and others have reported no significant change [140]. During diet-induced weight loss, the ghrelin level increases, potentially leading to weight regain [141]. Similarly, an increase in the ghrelin level following an AGB may be the result of weight loss due to the restrictive nature of this procedure, dietary limitations or a change in eating behavior [142,143]. This could explain why long-term sustained weight loss is not successful with this surgical approach.

BPD involves distal gastrectomy and the preservation of the gastric fundus, which still comes into contact with ingested nutrients. Some studies have shown no significant change in the ghrelin concentration post-operatively [144,145,146], while others have demonstrated an increase [147,148]. On the contrary, BPD-DS involves a VSG (with a resection of the gastric fundus). Hence, studies have described a fall in the ghrelin concentrations following this bariatric procedure [149]. Similarly, with laparoscopic VSG, the main source of ghrelin, the gastric fundus, is removed. Accordingly, several articles have demonstrated reductions in fasting and post-prandial ghrelin concentrations following VSG in both the short and long term [150,151,152,153,154,155].

Despite RYGB being one of the most effective treatments for obesity and obesity-related comorbidities, data on the inhibition of ghrelin release post-procedure remain controversial [156]. Several authors have described a decrease in ghrelin compared with levels in obesity and lean controls, which was sustained after 5 years post-operatively. This may explain the long-term effects of RYGB on weight loss [135,148,156,157,158,159]. Other studies have demonstrated either unchanged [153,160,161] or increased ghrelin concentrations post-RYGB [143,162], which suggests that ghrelin levels might not contribute to the reduction in food intake after RYGB. In exploring the cause of these diverse findings, consideration should be given to differences in surgical techniques (i.e., the size of the remaining gastric pouch and length of the alimentary limb); the lack of a standardized approach for vagal denervation (the absence of vagal impulses reduces ghrelin release); ongoing weight loss with a negative energy balance [133,148,156,163,164].

### 4.2. Cholecystokinin (CCK)

CCK is a peptide hormone secreted by the I cells of the duodenal mucosa, mainly in response to the presence of amino acids and fatty acids in the duodenum. CCK is responsible for stimulating the secretion of digestive enzymes from the pancreas, the contraction of the gallbladder leading to bile release and the slowing of gastric emptying [165,166]. Additionally, it activates the gut–brain axis and thereby induces satiety, controls energy homeostasis and lowers the glucose levels. CKK exerts its effects by binding to CCK1 and CCK2 receptors (also known as CCKAR and CCKBR, respectively), and it is widely distributed in the GI tract, CNS and peripheral neurons (including the NTS, hypothalamus and vagus nerve) [167,168,169,170]. It has been shown that the peripheral administration of CCK, which acts on CCK1 receptors, results in a dose-dependent initial reduction in dietary intake in rodents. However, this effect is not sustained in the long term, suggesting that a possible tolerance to CCK develops. Moreover, in rats, CCK appears to be associated with decreased meal sizes, but an increased number of meals leads to no change in total food intake [171,172]. Studies in humans have failed to demonstrate significant weight loss following the administration of CCK agonists, proving the limitations of using CCK as a therapeutic agent for weight loss [173].

The impact of bariatric procedures on the CCK level is an under-researched area. CCK secretion primarily increases after a mixed nutrient meal enters the duodenum. However, despite the duodenum being excluded from contact with nutrient contents post-RYGB, studies have demonstrated a rise in CCK secretion post-laparoscopic VSG and RYGB [158,174]. Peterli et al. assessed the effects of VSG and RYGB on the CCK levels and noted a significant increase following surgery, with a more prominent rise in the VSG group. This effect increased in magnitude from week 1 up to a 1-year follow up [158]. Possible explanations for the higher CCK secretion post-surgery may be the proliferation of CCK-secreting cells in the Roux and common limbs in RYGB or the activation of parasympathetic impulses and intraluminal releasing factors that stimulate CCK production [97].

### 4.3. Peptide Tyrosine–Tyrosine (PYY)

PYY_1-36_ is a 36 amino acid peptide that is produced post-prandially by the L cells in the distal small intestine and colon. Following cleavage in the circulation by the enzyme dipeptidyl-peptidase 4 (DPP-4), PYY_1-36_ is converted to its bioactive form, PYY_3-36_. The main effects of PYY_3-36_ are appetite suppression, a delay in gastric emptying, a reduction in post-prandial insulin secretion and alteration in colonic motility [175,176,177]. PYY_3-36_ elicits these effects by binding to Y2 receptors (Y2R) located in the vagus nerve, NTS and *POMC* in the ARC [175]. Both *AgRP*/*NPY* neurons and *POMC* neurons contain receptors for PYY, which suggests that these two areas of the CNS are vital for the regulation of food intake by PYY [178]. PYY leads to reduced *NPY* expression, increased c-Fos expression and the activation of *POMC* neurons, which is associated with an anorexigenic effect [175].

Le Roux and colleagues reported that the PYY_3-36_ level is lower after a meal in individuals with obesity than in normal-weight controls, suggesting a possible role of PYY_3-36_ deficiency in obesity. Furthermore, intravenous infusions of PYY_3-36_ in normal-weight men led to reduced food intake [179].

Several studies have shown that the post-prandial PYY_3-36_ level increases after bariatric surgery, regardless of the type of weight-loss surgery procedure undertaken (RYGB, VSG or AGB) [145,161,180]. However, in a recent study, Arakawa and colleagues reported that following VSG, the post-prandial PYY level rises to a lesser degree compared with RYGB, and despite an increase at 26 weeks post-VSG compared to baseline, the significance of this finding was not sustained at 52 weeks [181]. Other authors have also reported a more potent change in the PYY concentration post-RYGB, compared with following other bariatric procedures [143,150,182].

### 4.4. Glucagon-like Peptide 1 (GLP-1)

GLP-1 is a 30-amino-acid gut peptide that is produced and secreted by the L-EECs in the distal ileum and colon following a meal [183]. It is encoded by the proglucagon gene [184]. Its main functions are to stimulate post-prandial insulin secretion, delay gastric emptying, inhibit glucagon secretion and drive centrally mediated appetite reduction [185]. GLP-1 acts mainly by activating the GLP-1 receptors (family B of G-protein-coupled receptors) which are distributed in the CNS, GI system and pancreas [186]. Both the peripheral and central administration of GLP-1 led to the activation of neurons in the ARC, PNV, NTS and area postrema, inducing satiety [186,187]. The effects of GLP-1 on food intake extend beyond metabolic changes. Dickson et al. demonstrated that the activation of GLP-1 receptors also reduces food reward behavior by acting on the mesolimbic system, specifically on the ventral tegmental area and nucleus accumbens [188,189]. Additionally, GLP-1 exhibits incretin-like activity, potentiating glucose-dependent insulin secretion and inhibiting glucagon secretion by the islet cells. GLP-1 also stimulates pancreatic beta-cell neogenesis and suppresses their apoptosis [168]. In this context, GLP-1 may potentially be influenced by changes in aquaporin-7 expression, which has been noted to be downregulated after VSG in rats with obesity and may result in GLP-1-induced pancreatic steatosis improvement and insulin secretion [190,191].

Considering the physiological importance of GLP-1 in controlling islet cell function, appetite, inflammation and cardiovascular pathophysiology, the development of GLP-1 receptor (GLP-1R) agonists was a crucial step in obesity and diabetes treatment strategies [192]. There is abundant evidence to suggest that GLP-1 agonism reduces food intake and promotes weight loss [192,193]. In the US, there are now two GLP-1R agonists (Liraglutide and Semaglutide) approved for use in the treatment of obesity [194,195].

Multiple studies have reported considerably elevated post-prandial levels of GLP-1 following RYGB and SG [150,158,196,197,198,199]. A recent study found that BS procedures such as RYGB and SG may lead to the expansion of GLP-1-expressing cells in the rat and human gastric mucosa, as well as affecting the other D, X/A and enterochromaffin cells [200]. Furthermore, Dirksen et al. reported that the increase in the GLP-1 concentration following BS may be related to the patients’ weight loss outcomes, as individuals that achieved greater weight loss had higher GLP-1 levels in comparison to those with little weight loss at a 1-year follow-up [201]. Dar and colleagues noted a statistically significant, exaggerated GLP-1 response 10 years post-RYGB, indicating the durability of these changes [202]. Contrarily with AGB, a significant change in the GLP-1 concentration has not been observed [143,161,203,204]. In addition, it has been suggested that if vagotomy is performed during BS, the increase in GLP-1 is attenuated, as GLP-1 is thought to reduce appetite at the central level through vagal afferent nerve fibers [187].

### 4.5. Oxyntomodulin (OXM) and Glicentin

Both OXM and glicentin are proglucagon-derived peptides that are co-secreted from intestinal L cells together with GLP-1 and GLP-2 [205]. OXM is a 37-amino-acid peptide that structurally resembles glucagon with an additional intervening peptide-1 (IP-1) [206]. The main effects of OXM are the stimulation of insulin secretion, a reduction in the blood glucose concentration, the slowing down of gastric emptying and the suppression of gastric acid secretions [207,208,209]. Furthermore, OXM acts as an agonist of both glucagon and GLP-1 receptors. OXM only displays weak agonism to glucagon receptors mimicking its effect in the liver and pancreas [210]. However, OXM, by being a GLP-1 agonist, plays a more significant role in the reduction in appetite and food intake in rodents and humans [211,212,213,214]. The anorectic effects of OXM are discernible, even in mice lacking functional glucagon receptors, but they are absent in GLP-1R-negative mice, suggesting that OXM regulates food intake via GLP-1R [213]. Studies have also reported that the exogenous administration of OXM not only reduces food intake but also increases energy expenditure in humans [215,216].

Several studies have assessed the changes in the OXM concentrations pre-and post-BS [199,217,218,219]. None have reported a change in the fasting levels post-RYGB, SG or AGB; however, the post-prandial OXM concentration was found to increase following RYGB [199,217,219]. Furthermore, there has been recent progress in the development of OXM analogues as therapeutic agents for obesity and diabetes, with one analogue (LY3305677/IBI362) awaiting regulatory approval [220]. Some of the obstacles to the design of an effective OXM agent are rapid enzymatic inactivation of the molecule by dipeptydil peptidase 4 (DPP-4), as well as the achievement of a balance between the glucagon receptor and GLP-1R agonism to avoid the effect of hyperphagia [221].

The glicentin molecule contains the entire sequences of OXM (and thus also of glucagon) and glicentin-related pancreatic peptide (GRPP) [206]. Glicentin plays roles in the stimulation of insulin secretion and the inhibition of glucagon secretion, the reduction in gastric acid secretions, the regulation of gut motility and the promotion of gut growth [222,223,224,225]. Glicentin’s mechanism of action is still debatable, as its receptor remains unknown [226]. It has been shown that the hormone potentiates cyclic adenosine monophosphate (cAMP) production after binding to glucagon, GLP-1 and GLP-2 receptors [227].

Earlier studies have suggested that the degradation of the glicentin molecule into smaller fragments is essential to exert its action, which may limit its prospects as a potential therapeutic agent [220]. There is also very limited data on the impact of BS on the glicentin concentration. Nonetheless, elevated post-prandial glicentin levels after RYGB have been reported [217,219,226,228]. Poitou et al. explored the possible relationship between higher glicentin levels and post-prandial hypoglycemia in patients following RYGB [228]. Raffort et al. demonstrated a higher glicentin level post-RYGB as well as post-vertical-SG (VSG) with a more pronounced effect in the bypass group. They also discussed the idea that BS may restore the glicentin level to normal, as patients with obesity have been found to have lower glicentin levels than normal-weight individuals [229].

Furthermore, Nielsen and colleagues assessed the post-prandial responses of five gastrointestinal hormones (ghrelin, OXM, GLP-1, PYY and glicentin) before and 6 months after RYBG and VSG. Enhanced glicentin and OXM responses were predictive of greater weight loss and were associated with a decreased preference for energy-dense foods [219]. These results were replicated by Perakakis et al., suggesting that OXM and glicentin may have valuable roles as predictors of body weight loss following metabolic surgery. This could also aid in the identification of patients who may need additional support after bariatric procedures [199,226].

### 4.6. Neurotensin

Neurotensin is a 13-amino-acid peptide produced by the N-EECs predominantly located in the ileum. Its release leads to increased gastric and intestinal motility and increased fat absorption by stimulating the pancreatic and bile acid secretion [230].

Few studies have examined the neurotensin level in humans with obesity and the effect of neurotensin on appetite. The intracerebral and intraperitoneal administration of neurotensin in rodents has been reported to reduce food intake, whereas the anorexigenic effect of neurotensin in humans is still uncertain [231,232]. Cingoz and colleagues reported a higher neurotensin concentration in adolescents with obesity than healthy controls and no association with metabolic parameters, hyperphagia or food preferences [233]. Furthermore, the post-prandial neurotensin level increased more considerably post-RYGB compared with that following SG [180,234,235].

### 4.7. Gastric Inhibitory Polypeptide, Glucose-Dependent Insulinotropic Polypeptide (GIP)

GIP is a 42-amino-acid gut peptide synthesized by K cells, which are predominantly present in the duodenum and jejunum in response to nutrient ingestion. GIP acts directly on the GIP receptors (*GIPR*) expressed by pancreatic beta cells and stimulates insulin secretion [193,236]. GIP and GLP-1 are responsible for 70% of post-prandial insulin secretion [193]. In addition, GIP promotes triglycerides storage in the adipocytes through the stimulation of lipoprotein lipase [237]. Furthermore, *GIPR* is widely distributed in CNS regions involved in energy homeostasis [238]. Miyawaki et al. reported that *GIPR*-deficient mice had normal body weight when consuming a normal diet, and when challenged with a high-fat diet, they gained less weight and their insulin sensitivity was preserved [239]. Despite the obesogenic role of endogenic GIP, other studies have found that when utilized in the pharmacological context, GIP agonism has a weight-reducing effect similar to GIP antagonism [240]. One of the possible explanations for this paradox is that chronic GIP agonism leads to the desensitization of *GIPR*, ultimately resulting in antagonism [240]. Studies have suggested that this effect may occur at the CNS level. For example, Adriaenssens and colleagues observed *GIPR* expression in the ARC, in the DMN and PVN in the hypothalamus, which play major roles in energy homeostasis [241]. A recent study by Zhang et al. demonstrated the suppression of food intake and reduced body weight in diet-induced obese mice following chronic central and peripheral administration of acyl-GIP agonist [242]. These results demonstrate the potential of GIP agonists to be used as therapeutic agents to fight obesity.

Contrarily, the reported changes in the post-prandial GIP levels following bariatric procedures such as RYGB and SG are inconsistent. Some studies have reported a reduction following RYGB and VSG [199,243], whilst others have described either an increased postprandial concentration [218,244] or no significant change [185,217,245]. In contrast, AGB studies consistently reported no change in fasting or post-prandial GIP levels [199,204,243,246].

### 4.8. Gastrin

Gastrin is a family of multiple peptides with biological activity. It is secreted by the G cells in the gastric antrum, duodenum and pancreas. Release appears to be enhanced when nutrients are in direct contact with the G cells. Gastrin’s main role is to stimulate gastric acid (HCl) production by the parietal cells in the stomach and increase gastric motility [165,184].

During bariatric procedures such as RYGB, the gastric antrum and duodenum are excluded from contact with nutrients which could lead to a decrease in the gastrin level post-operatively. Several studies have reported lower gastrin levels in individuals that have undergone RYGB compared with controls, and these changes were sustained at 1 year post-surgery [163,247,248]. Moreover, Grong and colleagues described a fall in the gastrin level only in the RYGB group, while there was no significant change in the VSG group [248]. Stenstrom and colleagues assessed the role of a low gastrin level following gastric bypass and its association with weight loss. They observed body weight, the thickness of the oxyntic mucosa, the serum gastrin levels and enterochromaffin-like cells (ECL cells) activity in mice after gastric bypass alone and after gastric bypass with gastrin infusion. Weight loss was more pronounced in the gastric bypass alone group, and this was likely related to hypergastrinemia and reduced ECL activity in the gastric mucosa following gastric bypass [249].

### 4.9. Fibroblast Growth Factors (FGFs)

FGFs are a family of 22 proteins responsible for cell growth and differentiation, development, angiogenesis, wound repair and metabolism [250]. Whilst the majority of FGFs have autocrine or paracrine functions, FGF19 and FGF21 are hormone-like and act as endocrine factors [251].

FGF15 is the murine ortholog of FGF19 [251]. FGF19 is produced in the ileum, gallbladder and brain and plays a role in the regulation of glucose and lipid metabolism, energy expenditure and body adiposity [250,252]. FGF15/19 transcription in the ileum is regulated by bile acids through interaction with the nuclear farnesoid X receptor (FXR) [252]. FGF15/19 expression increases with the elevation of bile acids in the distal ileum following a meal [253,254]. Multiple studies have reported increased FGF19 levels following bariatric procedures such as RYGB and SG [255,256,257,258,259,260]. This rise has not been observed following diet-induced weight loss [255]. Results after AGB are more inconsistent. Escaleri et al. reported a decrease in the FGF19 level after laparoscopic AGB, whereas Thoni et al. described a continuous increase post-operatively [257,261]. A recent meta-analysis by Ryan et al. established that the FGF19 level significantly increased after VSG, RYGB and duodenojejunal bypass, with AGB failing to achieve the same effect, while BPD led to a decrease in the FGF19 level [262].

FGF21 is predominantly synthesized in the liver in response to starvation, the consumption of a ketogenic or high-carbohydrate diet or the administration of fibrate drugs. It is also expressed in white and brown adipose tissue, where it is induced by fasting/refeeding and cold, respectively [263,264]. FGF21 acts on FGF receptors which are expressed widely, in complex with the β-Klotho co-receptor, which is present in WAT, BAT, liver as well as in the hypothalamus (suprachiasmatic nucleus, NTS, area postrema and nodose ganglia). It has been suggested that FGF21 acts on the CNS level given its ability to cross the blood–brain barrier and the presence of β-Klotho receptors in the brain [265]. However, Lan et al. compared the tissue-specific mechanisms underlying the actions of FGF19, FGF21 and FGF21 mimetic antibodies in mice. They discovered that all three molecules require β-Klotho in neurons, but the exertion of their metabolic effects on body weight or glycemia in hepatocytes and adipocytes was found to be independent of the presence of β-Klotho receptor [266]. Furthermore, Owen and colleagues demonstrated that FGF21 stimulates sympathetic outflow at a central level, which in turn increases energy expenditure and potentiates weight loss in diet-induced obese mice [263]. FGF21 also acts to increase fatty acid oxidation and improve insulin sensitivity. FGF21 levels are increased in patients with obesity, which suggests that obesity is a state of FGF21 resistance [250]. The effect of BS on the FGF21 is still an under-researched area. Gomez-Ambrosi and colleagues demonstrated that FGF21 concentration was decreased after diet-induced weight loss and post-SG, but no change was noted post-RYGB [255].

### 4.10. Bile Acids

Primary bile acids are synthesized from cholesterol in the liver. Following conjugation with taurine or glycine, they are secreted into bile and expelled into the small intestine after a meal to aid the absorption of fat and fat-soluble vitamins. Subsequently, they are absorbed in the distal ileum and recycled back into the liver [267]. Bile acids play a significant role in glucose and lipid metabolism and energy homeostasis, which extends further than facilitating the absorption of dietary lipids.

There is complex crosstalk between bile acids, gut hormones and the microbiome. Bile acids promote GLP-1 secretion by activating G-protein-coupled receptors (TGR5) located in L cells [268]. Additionally, TGR5 receptors are present in skeletal muscle and brown adipose tissues, and their activation leads to increased energy expenditure through the increased conversion of inactive thyroxine T4 to its active form [267]. Furthermore, bile acids are ligands for the nuclear farnesoid X receptor (FXR), which has various effects on metabolism. The binding of bile acids on FXR in pancreatic cells leads to increased insulin release, whilst binding at intestinal cells stimulates the secretion of FGF19 which suppresses gluconeogenesis, promotes glycogen and protein synthesis and increases energy expenditure and the metabolic rate, leading to weight loss [269,270,271]. Moreover, bile acids protect the gut from epithelial deterioration and bacterial translocation through a direct antimicrobial effect, by limiting the expansion of the microbial population within the GI tract, and by stimulating the expression of antimicrobial defense genes via FXR activation [272,273].

The association of bile acids with the CNS occurs through direct and indirect pathways. Direct CNS signaling is elicited by binding to FXR and TGR5 in the brain, whereas the indirect pathway involves the activation of FXR/TGR5 in the gut, which then stimulates the release of FGF19 and GLP-1, which both act at a central level [274]. Moreover, there are multiple nuclear receptors activated by bile acids such as the pregnane X receptor (PXR/NR1H2), the Vitamin D receptor (VDR/NR1H1), the CAR (NR1H3) and the glucocorticoid receptor [275]. Bile acids also regulate cell membrane receptors in the CNS, such as sphingosine 1 phosphate receptor 2, muscarinic receptor M2 and M3, N-methyl D aspartate receptor and gamma-aminobutyric acid A receptor [276].

Following both RYGB and SG, the circulating levels of bile acids increase considerably. These serum levels are negatively correlated with post-prandial blood glucose and positively correlated with the GLP-1 level, which indicates that the production of bile acids has a positive impact on glucose and lipid metabolism post-BS [277,278,279,280]. The causality of increased bile acids levels after surgery is not clear. One of the possible explanations is the re-routing of bile acids to the mid-jejunum in RYGB, which would explain the most consistent increase in bile acids reported following bypass surgery in comparison with following SG [218,281]. Another hypothesis is an adaptive increase in bile acids as a result of lipid malabsorption to compensate for higher volumes of bile acids escaping the enterohepatic circulation [280].

### 4.11. Secretin

Secretin is a 27-amino-acid gut peptide that is produced by the S cells in the duodenal mucosa in response to a low intraluminal pH. Secretin’s main functions are the inhibition of gastric acid secretion, the stimulation of bicarbonate production from the pancreas, the promotion of bile acid production in the liver and the inhibition of gastric emptying [282]. In addition to its gastrointestinal effects, secretin has metabolic effects such as the induction of lipolysis and enabling reductions in food intake [283,284]. Cheng et al. found that secretin’s anorexigenic effect is mediated through secretin receptors in the vagal sensory nerves and melanocortin signaling in the CNS [284]. Secretin also mediates the gut–BAT–brain axis, promoting satiety through meal-induced thermogenesis in mice and humans [285]. There is limited and inconsistent evidence of changes in the secretin levels after BS. Nergard and colleagues assessed the villi length and density of secretin in individuals undergoing jejunal biopsies at baseline and 12 months after RYGB and found no significant change post-operatively [286]. On the contrary, Rhee and colleagues reported a reduced density of cells immunoreactive for secretin post-RYGB in patients with diabetes compared with age- and BMI-matched controls [287].

### 4.12. Nesfatin

Nesfatin-1 is an 82-amino-acid peptide derived from *nucleobindin-2* mRNA, which is produced by different areas of the brain. Nesfatin-1 is released from the gastric and intestinal EEC and pancreatic cells and is able to cross the blood–brain barrier. Hence, nesfatin-1 exerts central and peripheral effects such as reductions in appetite and food intake, a delay in gastric emptying, the promotion of glucagon and insulin secretion, the improvement of insulin sensitivity and a reduction in the blood glucose concentration [288,289,290].

Following BPD-DS, the nesfatin level was found to be reduced compared with controls [291]. Chen et al. discovered that the reduced level post-RYGB was likely related to the vagotomy performed during the procedure [288]. Majorczyk et al. found a non-statistically significant reduction in the levels post-RYGB and SG [292], while Wei-Jei Lee et al. found a significantly reduced nesfatin-1 levels post-SG and RYGB [293].

### 4.13. Gustducin

Gustducin is a gustatory G-protein that is expressed in taste receptor cells as well as being present in a limited subset of EECs in the stomach and small intestine [294]. It has been suggested that alpha-gustducin plays a role in gut hormone release as well as in gustatory function [295]. Studies of rodents have linked functional intestinal nutrient sensing through alpha-gustducin with GLP-1 secretion following RYGB [296]. Sweet taste receptors in L cells are coupled with alpha-subunit alpha-gustducin, and their activation stimulates GLP-1 secretion [296]. Steensels et al. studied the role of gustducin-mediated signaling in metabolic improvement and intestinal adaptations after RYGB in wild-type and alpha-gust mice. In wild-type mice, alpha-gustducin increased L-cell differentiation (foregut) and the L-cell number (foregut and hindgut). In alpha-gust mice, the effect on gut hormone levels was thought to be due to increased peptide sensor and glucose transporter expression in the Roux limb as well as increased cecal butyrate and propionate levels and their subsequent activation of free fatty acids receptors [295]. Furthermore, alpha-gustducin was found to stimulate the expression of glucose transporter Glut 2, leading to the increased absorption of oral glucose [294].

### 4.14. Uroguanylin

Uroguanylin is a 16-amino-acid satiety peptide that is secreted as a pro-hormone (pro-uroguanylin) from duodenal EECs. Pro-uroguanylin undergoes post-prandial enzymatic conversion to its active form [297]. Uroguanylin binds to the guanylate cyclase 2C (GUCY2C) transmembrane receptor. Analogues to GUCY2C are responsible for decreasing water and sodium permeability and increasing chloride secretion in the gut. The overproduction of uroguanylin can lead to acute diarrhea [298].

In the hypothalamus, pro-uroguanylin is converted to uroguanylin, inducing GUCY2C signaling, which activates the neuropeptide *POMC*, suppressing appetite at the central level [299]. This suggests that the central administration of uroguanylin may be an effective treatment for obesity [300]. Patients with obesity were found to have a considerable decrease in pro-uroguanylin after a meal in comparison with lean individuals, suggesting that obesity is associated with an impaired post-prandial pro-uroguanylin response [301]. Studies have demonstrated increased serum levels of uroguanylin following metabolic surgery [302]. Furthermore, Torquati and colleagues found elevated post-prandial levels after RYGB, but this was reduced in the fasted state. There was also no correlation observed between circulating pro-uroguanylin levels and hunger perception before and after RYGB. The authors concluded that the pro-uroguanylin–uroguanylin–GU2CYC system does not play a role in the anorexigenic effect after RYGB [301].

### 4.15. Obestatin

Obestatin is a 23-amino-acid peptide that is mainly produced in the stomach but also in other tissues. Its biological effects with regards to food intake remain controversial; however, obestatin has multiple functions, including the regulation of cell proliferation, the regulation of glucose and lipid metabolism and anti-inflammatory actions [303]. Obestatin was initially considered an anorexigenic hormone that inhibits the action of ghrelin; however, studies have reported that it does not suppress food intake in the absence nor the presence of ghrelin [304,305,306]. The plasma concentrations of obestatin were found to be lower in individuals with obesity compared with lean ones [307]. However, the relationship between obestatin and BS is debatable. Several authors have reported an increase in obestatin post-RYGB, whilst other studies have failed to detect any difference in concentration pre-and post-operatively [308].

### 4.16. Glucagon-like Peptide-2 (GLP-2)

GLP-2 is a 33-amino-acid peptide that is co-secreted with GLP-1 by the L-EECs in response to nutrient ingestion. GLP-2 has a role in stimulating gut hypertrophy via ileal cell hyperplasia and reducing apoptosis. It has been used in the treatment of patients with short gut syndrome [309,310]. GLP-2 receptors (GLP-2R) are expressed in EECs and pancreatic alpha cells as well as in neurons, such as enteric, vagal sensory and central neurons [311,312,313,314,315]. Guan et al. suggested that GLP-2 is a key neuroendocrine factor that plays a role in the control of feeding behavior and glucose homeostasis via the activation of GLP-2R in *POMC* neurons which are present in the hypothalamus. They demonstrated that mice with GLP-2R deletion exhibited hyperphagia, accelerated gastric emptying, glucose intolerance and hepatic insulin resistance [315]. Similarly, Sun and colleagues found that GLP-2 microinjections in the NTS suppressed food intake in fasted refeeding rats but did not affect free-feeding rats. Hence, GLP-2 appears to inhibit food intake, and this is mediated by *MC4R* in the NTS [316].

Several studies have assessed the GLP-2 level following RYGB and observed a significant increase post-operatively [317,318]. Romero et al. described an increase in the GLP-2 concentrations after both RYGB and SG [319]. Dimitriadis and colleagues suggested that this may be related to weight stabilization, the late reduction of diarrhea and malabsorption and the minimization of the consequences of bacterial overgrowth [165].

### 4.17. Leptin

Leptin is a hormone (specifically an adipokine) that plays an important role in satiety and food intake regulation, and its levels correlate with fat mass and adiposity [320]. Furthermore, leptin levels have a circadian rhythm and are highest during the nighttime [321]. In the obese state, this rhythm is disrupted, and leptin levels are high overall. Leptin secretion is stimulated by intracellular glucose metabolites and circulating insulin, but it is inhibited by leptin signaling in hypothalamic *POMC* neurons during fasting [322]. Leptin is produced primarily in adipose tissue but it has also been found to be secreted from gastric mucosa (gastric EECs) in lesser amounts [323]. Since leptin is considered an adipokine, we only briefly discuss its neuroendocrine mechanisms in this review.

Leptin acts on the leptin receptors which are widely distributed in the CNS and has an effect on energy homeostasis as well as neuroendocrine and immunological function [324]. Leptin acts primarily in the hypothalamus, inhibiting the orexigenic *AgRP*/*NPY* and stimulating anorexigenic *POMC* neurons in the ARC, leading to the suppression of energy intake and increasing energy expenditure [325]. Moreover, studies have suggested that the CNS leptin–melanocortin system, which activates *POMC* and *MC4R*, can also normalize glucose levels independent of insulin action, the ANS or the pituitary–adrenal–thyroid axis [326]. This suggests that once the mechanisms of the leptin–melanocortin system are unraveled, new therapeutic strategies to tackle both obesity and diabetes may be on the horizon. In addition, leptin appears to play a role in immunity and inflammation, and its deficiency is associated with dysregulated cytokine production. Circulating leptin has a protective effect on the host, leads to the stimulation and activation of monocytes in vitro and increases neutrophil chemotaxis [327].

Data on leptin levels post-BS are limited, and the results are controversial. Some studies have demonstrated reductions post-AGB, RYGB and BPD [137,328,329,330]. Furthermore, Edwards and colleagues found that the downregulation of leptin expression was greater in patients who lost more excess body weight and was more pronounced following RYGB compared with AGB [331]. Conversely, other studies suggested that following RYGB and VSG, leptin concentration is no lower than pre-operative values [218].

Apart from leptin, there are several other adipokines and factors which include hepatokines and myokines that are related with obesity and are affected by BS such as adiponectin, Insulin-Like Growth Factor Binding Protein 2, sex hormone-binding globulin and fetuin A [332,333,334]. However, a detailed presentation of the role of these factors in obesity and the effects of BS is outside the scope of our review.

### 4.18. Insulin

Insulin is a peptide hormone that is expressed by the β-pancreatic cells in response to blood glucose [335]. Insulin is a powerful metabolic hormone that is involved in glucose, fat and protein metabolism and has an established action on peripheral tissues and organs. Its glucose-lowering effect involves the inhibition of glycogenolysis and gluconeogenesis, the increase in transport of glucose into fat and muscle and the stimulation of glycogen synthesis [336]. Furthermore, insulin acts on the CNS by binding to insulin receptors which are highly expressed in the hypothalamus, cerebellum, cortical and subcortical regions [337]. Kullmann et al. reported that central insulin action leads to the enhancement of whole-body insulin sensitivity and suppresses the production of endogenous glucose. Insulin curbs food intake, reduces the rewarding properties of energy-rich food and increases cognitive control through its action on the mesocorticolimbic circuitry [337]. These mechanisms may be impaired in individuals with obesity.

BS alters insulin secretion and sensitivity and contributes to the improvement or remission of type 2 diabetes post-operatively. Circulating insulin level is defined by a balance between insulin secretion and insulin clearance. Insulin clearance is reduced in obesity and type 2 diabetes, which leads to a state of hyperinsulinemia [338]. Following RYGB, post-prandial insulin secretion is increased with an exaggerated response to oral glucose due to the insulinotropic effect of nutrient-activated incretin hormones such as GLP-1 and GIP [339,340], whereas fasting insulin is significantly reduced after RYGB [341].

Improved insulin sensitivity plays a major role in the normalization of glucose levels post-operatively. Mingrone et al. reported that the improvement of whole-body insulin sensitivity is dependent on the length of bypass of the proximal intestine, with BPD demonstrating a greater effect than RYGB [342]. This occurs in the early post-operative period before any significant weight loss has taken place [342]. The production of anti-incretin hormones which induce insulin resistance could be inhibited, as BS avoids the passage of nutrients through the proximal small bowel [343]. Additionally, Salinari et al. demonstrated that bypassing the duodenum and proximal jejunum reverses insulin resistance in both diabetic subjects and subjects with normal glucose tolerance [344].

A summary of the alterations in gut peptides after BS is shown in Table 1.

## 5. Gut Microbiota

The bacterial populations that colonize the neonatal gut soon after birth vary but become relatively stable after the second year of life [346,347]. The gastrointestinal tract harbors more than 10^14^ bacteria, the vast majority of which reside in the large intestine [348]. The intestinal microbiota is mainly composed of the phyla *Firmicutes, Bacteroidetes, Proteobacteria, Actinobacteria, Verrucomicrobia* and *Fusobacteria* [349,350]. There is large inter-individual variability in the composition of gut microbiota, but there is also a core microbiota composition that is common for many individuals [351].

The multiple roles of the gut microbiota include energy harvest from dietary intake, metabolism regulation, the modulation of the immune response and protection against intestinal pathogens and the control of epithelial cell proliferation and differentiation [352,353,354]. The disruption or alteration of the normal balance of the gut microbiota, which is referred to as dysbiosis, is associated with the dysregulation of the immune system, inflammation and various diseases, including T2DM and obesity [355,356,357,358]. A pioneering metagenome study by Wang et al. showed that gut dysbiosis was correlated with numerous T2DM-associated markers, suggesting that microbiome is important in the development of T2DM [359]. Studies involving mouse models have suggested that the gut microbiota not only contributes to the development of obesity but also to the complications associated with excessive body fat accumulation [360,361,362]. Obesity is characterized by reduced bacterial diversity and functional disturbances of related metabolic pathways. Up to 75% of patients with severe obesity, suitable candidates for BS, display low microbial gene richness which is associated with increased trunk-fat mass and metabolic co-morbidities including T2DM and hypertension [362]. Moreover, diet seems to play a key role in the composition and function of the gut microbiota [357]. The consumption of a high-fat/high-carbohydrate diet that leads to weight and fat gain induces changes in the gut microbiota with a higher relative abundance of *Firmicutes*. In contrast, the consumption of a diet low in carbohydrates and fat leads to decreases in weight gain and obesity with a higher relative abundance of *Bacteroides* [363].

Emerging evidence suggests that the disruption of the gut microbiota may affect weight gain through several mechanisms. These mechanisms include energy harvest, the generation of short-chain fatty acids (SCFAs), the establishment of satiety through the gut–brain axis, the modification of the inflammatory responses of the host and the regulation of genes promoting the storage of fat in the adipose tissue [364,365,366]. Dietary polysaccharides and proteins that are not digested in the small bowel undergo bacterial fermentation and hydrolysis in the large bowel via the gut microbiota, producing SCFAs and gases [349,367]. This process of energy extraction in the form of SCFAs accounts for approximately 10% of the host’s daily energy requirements [368]. Increased levels of SCFAs in feces are found in individuals with obesity and are related to changes in the microbiota composition [122,366,368]. On the other hand, some studies have found that increased fermentation by the gut bacteria is protective against the development of obesity [349,369]. Some studies have demonstrated that energy harvest and SCFA production are not necessarily associated with weight gain but that some SCFAs have beneficial roles in the host’s metabolism and energy regulation [122]. Specifically, SCFA administration is correlated with an enhanced metabolic state, a reduction in the consumption of food and a reduction in body weight via the activation of gut hormone synthesis [370,371].

Moreover, SCFAs act as signaling molecules that establish communication between the gut and the brain by stimulating G-protein-coupled receptors (GPCRs) located throughout the GI tract, specifically in L cells, to produce and release hormones such as PYY and GLP-1, which are anorexigenic [354,372,373,374]. The GPCRs that are bound to and activated by SCFAs are free-fatty-acid receptors 2 and 3 (FFAR2/FFAR3), which are not only located in the EECs, particularly the L cells of the GI tract, but are also expressed within the PNS [366,372]. Upon their activation in the SNS, they regulate storage mechanisms within the adipose tissue and stimulate muscle and liver cells to utilize glucose [366,375]. In addition, specific gut bacteria, such as *Akkermansia muciniphila, Faecalibacterium prausnitzii, Bifidobacterium* and *Lactobacilli*, are associated with improvements in the gut barrier function through the GLP-2 mediated pathway and stimulation of the endocannabinoid system [169,376]. These changes enhance intestinal cell growth and function and the differentiation of EECs and increase the concentration of hormones including GLP1, GIP and PYY, which increase satiety and decrease food intake and adiposity [376].

Normally, the gut microbiota protects the integrity of the mucosal barrier [9,39]. Obesity, metabolic syndrome and the consumption of a high-fat diet, leading to the disruption of the gut microbiota, result in the reduced expression of tight junction proteins and increased gut permeability. Subsequently, mucosal inflammation and bacterial translocation lead to the leakage of lipopolysaccharides (LPS) and bacterial metabolites (SCFAs and trimethylamine N-oxide (TMAO)) into the circulation and metabolic endotoxemia [377,378,379]. Finally, this reaction induces systemic low-grade inflammation with further metabolic consequences, including insulin resistance and obesity [380]. The systemic increase in LPS and the subsequent activation of Toll-like receptor (TLR4) results in the synthesis of inflammatory cytokines, white adipose tissue inflammation, reduced insulin sensitivity and changes in the gut microbiota [381,382]. Another effect of LPS is inhibition of the interstitial cells of Cajal, which leads to an alteration om neurotransmitter release in the ENS and the release of gut hormones [383].

The gut microbiota can directly communicate and alter CNS pathways. This has been demonstrated by studies assessing neurochemical changes following the administration of probiotic treatments. Specifically, increases in *Bifidobacterium* and *Lactobacilli* have been associated with increased vagal stimulation and reduced LPS-induced inflammation, which enhances gut barrier function and potentiates SNS activity. Additionally, gut microbiota alterations may modulate signals produced from CNS neurotransmitters such as serotonin [384]. The above changes in the microbiota influence, directly and indirectly, the CNS and are associated with decreased anxiety and stress in patients with irritable bowel syndrome, decreases in food cravings and depression and increases in satiety and self-esteem [122,385,386,387,388]. It is worth mentioning that gut microbes can alter the gut–brain homeostasis by influencing the metabolism of tryptophan (Trp) through the kynurenine (Kyn) degradation pathway [389,390]. Tryptophan and its metabolites are essential for the development of the CNS and ENS and are key players in regulating several physiological processes such as emotions, hunger, colonic motility and gut secretory activity [389,390].

### Gut Microbiota and Bariatric Surgery

Several studies have shown that the gut microbiota is significantly altered following BS, which may directly contribute to the observed reduction in adiposity [391,392]. The microbiota composition after BS is not only affected by digestive tract modifications but also by decreases in systemic and adipose tissue inflammatory responses [393]. The changes in the composition and function of the gut microbiota occur soon after surgical interventions even from the first week [391]. The most common change reported is a decrease in the abundance of *Firmicutes* (e.g., *Lactobacillus, Faecalibacterium prausnitzii* and *Coprococcus comes*) and increases in *Bacteroides* and *Proteobacteria* (*Escherichia* and *Klebsiella*) [391,394,395]. In addition, the increased gut microbiota diversity observed post-BS is associated with an increase in the *phyla Verrucomicrobia* (e.g., *Akkermansia muciniphila*) and *Fusobacteria* and a decrease in *Actinobacteria* (e.g., *Bifidobacterium*) [391,394,395]. The high concentrations of *Proteobacteria* and *Bacteroides* after BS are associated with decreased systemic inflammation, improved glucose homeostasis and weight loss. On the other hand, the reduction in the *Firmicutes* concentration does not allow increased caloric absorption from energy sources and halts further weight gain [396,397,398].

BS not only leads to caloric restriction but also causes anatomical rearrangements of the GI tract with subsequent changes in the nutrient transit time. These changes further contribute to modifications of intestinal hormones and bile acids and restructure of the gut microbiota [391,399,400]. The anatomical rearrangements of the GI tract resulting in increased luminal pH of the stomach and distal gut following surgery lead to bacterial overgrowth and changes in the bacterial populations. The decreased acidity favors the growth of *Escherichia coli, Akkermansia muciniphila* and *Bacteroides* species [394]. The abundance of *Akkermansia muciniphila* increases after RYGB, and this is negatively associated with obesity and positively associated with decreased inflammation and increased insulin sensitivity [376,401]. Several mechanisms by which *Akkermansia muciniphila* achieves the above-mentioned metabolic results have been described. Firstly, this species helps to maintain the intestinal mucosal barrier through its involvement in mucin degradation. Secondly, it reduces the levels of circulating LPS, causing a reduction in metabolic endotoxemia. Lastly, it increases the number of L cells that secrete GLP-1 and GLP-2. The increased abundance of *Akkermansia muciniphila* following BS is correlated [402,403] with T2DM remission [402,403]. The abundance of *Escherichia coli* increases after RYGB, and this is inversely correlated with weight loss and the leptin level [404]. The increased proportions of *Bacteroides* post-RYGB are directly related to a greater reduction in body fat mass and leptin [404].

Different bariatric procedures result in different microbiota-related outcomes. This can be explained by the different rearrangements of the GI tract caused by each procedure. Specifically, the most significant changes in the gut microbiota are observed following RYGB, as this technique alters the GI tract anatomy to a great extent [405,406]. Ilhan et al. showed that microbial diversity in the gut was higher in a group of patients who underwent RYGB compared with those who underwent laparoscopic AGB. An abundance of *Escherichia, Veillonella* and *Streptococcus*, along with an abundance of amino acid and carbohydrate fermentation products [407] was observed. These differences were attributed to the profound changes in the gastrointestinal anatomy caused by the RYGB [407]. Following RYGB, there is an alteration of bile flow due to the anatomical rearrangement which creates an environment that stimulates the growth of bile-acid-transforming bacteria. The structural changes also lead to the growth of facultative anaerobes, and this is associated with the increased microbial diversity following RYGB [407]. According to Medina et al., RYGB and VSG alter the gut microbiota differently. Specifically, an increase in *Proteobacteria* was observed six months after both RYGB and VSG, while *Bacteroidetes* increased in RYGB but decreased in the VSG group [408]. Recently, the Trp/Kyn pathway, a route commonly affected by gut microbiome alterations, has been recognized as a novel mechanism related with obesity and BS. Transcriptomic analysis before and after VSG in mice by Bernard et al. identified several biomarkers which appeared to participate to the Trp metabolic pathway [409]. Additionally, this study showed that alterations in the Kyn pathway were associated with changes in the taste of fat in human subjects, suggesting a role in fat taste sensitivity via the CNS [409].

There is evidence that BS induces changes in the gut microbiota composition, and these changes appear to be associated with food choices [410]. Following BS, a food shift in intake away from energy-dense food such as high-fat and high-sugar products and a preference for healthier options has been observed [411]. The root of this behavior can be multifactorial, reflecting either a conscious avoidance or assumed responsibility or even changes in taste and palatability following bariatric surgery [100,412]. Dietary preferences are different for each individual after BS; therefore, various diets can affect the gut microbiome differently [411]. For instance, the typical “Western diet”, which is rich in fat, is associated with the *Bacteroides* enterotype and usually decreases after BS due to a dietary shift towards healthier choices [410]. A Mediterranean diet is associated with a decreased abundance of *Prevotella copri* and reduces the risk of cardiometabolic disease [413]. In addition, the use of probiotic supplementation after BS has shown a beneficial modulation in the gut microbiome profile, a promotion of the absorption of micronutrients and an improvement in inflammatory markers; however, these changes seem to be transient [410,414]. Gut microbiome composition is important for the metabolic benefits observed after BS, and maintaining a healthy nutritional status is vital after BS, as it has been found that microbiome abundance is decreased in nutrient deficiency often caused by BS [415]. Recently, Quilliot et al. highlighted the best practice for nutritional care and the need for adequate vitamins (such as D and B12) and mineral supplementation after BS [416].

## 6. Conclusions and Future Perspectives

Currently, BS is considered the most effective treatment for obesity, especially for patients with severe obesity. BS results in sustained weight loss as well as inducing beneficial metabolic effects and improving many obesity-related comorbidities, for example, such as remission from T2DM [417]. In addition, BS has been found to be safe, with morbidity rates and mortality risk similar to those of commonly performed procedures, while the risks of severe obesity outweigh those of BS [418]. This review has summarized the available data regarding the neuroendocrine mechanisms and neurohormonal alterations that occur in the gut–brain axis following BS. Alterations in the CNS neuropeptides (*AgRP*/*NPY*, *POMC*, *BDNF*, etc.), gut hormones (CCK, PYY, GLP-1/2, OXM, etc.), bile acid metabolism and gut microbiota after BS appear to impact appetite regulation and satiety energy balance, thereby affecting weight control and metabolism.

However, despite the beneficial metabolic outcomes, BS remains underutilized, with less than 1% of eligible patients with obesity undergoing bariatric procedures [419,420]. Additionally, the metabolic benefits and their mechanisms are still not fully understood, and current evidence has mostly been derived from in vitro and in vivo studies [184]. BS is no longer characterized as only a weight-loss procedure. Instead, the term “metabolic surgery” has been proposed, given the multiple and profound metabolic effects of BS on the human body that occur through mechanisms beyond weight loss [417]. The development of novel non-invasive therapies with sustainable results that mimic the effects of BS is a promising and rapidly evolving field [27]. To achieve this, a better understanding of the mechanisms associated with the observed metabolic benefits of BS is considered crucial. Therefore, more studies are needed to further elucidate the underlying pathophysiological pathways and the overall spectrum of metabolic effects associated with bariatric (metabolic) surgery.

## Figures and Tables

**Table 1:** Gut peptides and their alterations after BS.

Peptide	Site of Secretion	Effect on Food Intake	Main Functions	Modulation after BS	References
Ghrelin	P/D_1_ cells (gastric fundus)	↑	-↑ food intake	↑ AGB ↓ BPD-DS+VSG (-) RYGB	[127,128,129,130,132,133,135]
Gastrin	G cells (pyloric antrum of stomach, duodenum and pancreas)	↓	-↑ HCl production -↑ gastric motility	↓ RYGB (-) VSG	[152,163,184,247]
Leptin	Adipose tissue and gastric EECs	↓	-↓ glucose production and steatosis in the liver -↑ glucose uptake and fatty acid oxidation in muscles -↓ insulin and glucagon secretion -↑ sympathetic nervous system tone -↑ thyroid hormones-modulates immunity and fertility	(-) RYGB, VSG	[218,323,324,328]
Obestatin	Stomach	?	-controversial role in food intake -regulates cell proliferation and survival -regulates glucose and lipid metabolism	(-) RYGB	[303,304,305,306,307]
Nesfatin	EECs (stomach and small intestine), pancreatic cells	↓	-↓ appetite -stimulates glucagon and insulin secretion -improves insulin sensitivities	↓ RYGB ↓ VSG ↓ BPD-DS	[288,289,290,291,292,345]
Gustducin	EECs (stomach and intestine)	?	-stimulates GLP-1 secretion -promotes absorption of oral glucose	?	[294,295,296]
CCK	I cells (duodenum)	↓	-stimulates secretion of digestive enzymes from pancreas -stimulates release of bile from gallbladder -slows down gastric emptying -induces satiety	↑ RYGB ↑ VSG	[152,158,166,174]
Secretin	S cells (duodenum)	?	-inhibits secretion of gastric acid -stimulates production of bicarbonate -stimulates bile production	(-)/↓ RYGB	[282,286,287]
Uroguanylin	EECs (duodenum)	↓	-induces satiety -↓ water and sodium permeability and chloride secretion in the gut	↑ RYGB	[297,298,300,302]
GIP	K cells (duodenum and jejunum)	?	-stimulates insulin release -promotes triglyceride storage in the adipocytes	(-) RYGB (-) VSG (-) AGB	[199,218,243,244]
GLP-1	L cells (distal ileum and colon)	↓	-stimulates post-prandial insulin secretion -delays gastric emptying -inhibits glucagon secretion -↓ appetite centrally	↑ RYGB ↑ VSG	[150,158,183,185,196]
GLP-2	EECs in small intestine	↓	-stimulates gut hypertrophy -↓ apoptosis	↓ RYGB ↓ VSG	[309,310,317,318,319]
Glicentin	L cells (ileum)	?	-may stimulate insulin secretion, gastrointestinal motility and gut growth	↑ RYGB	[217,219,226]
Neurotensin	N cells (ileum and CNS)	↓	-inhibits intestinal and gastric motility -↑ fat absorption by increase in pancreas and bile acid secretion	↑ RYGB ↑ VSG	[180,230,231,232,233,234,235]
OXM	L cells (ileum)	↓	-↓ food intake -↓ glucose levels -↓ gastric acid secretion and delays gastric emptying -↑ energy expenditure	↑ RYGB	[184,211,213,217,219,226]
PYY(3-36)	L cells (distal ileum and colon)	↓	-delays gastric emptying -↓ appetite centrally -↓ post-prandial insulin production -alters colonic motility	↑ RYGB ↑ VSG ↑ AGB	[161,175,176,180]
FGF15/19	Ileum, gallbladder and brain	↓	-suppresses bile acid synthesis and gluconeogenesis -promotes glycogen and protein synthesis -↑ energy expenditure	↑ RYGB ↑ VSG (-) AGB	[255,257,258,259,261,271]

AGB: adjustable gastric banding, BPD-DS: biliopancreatic diversion with duodenal switch, CCK: cholecystokinin, EECs: enteroendocrine cells, FGF: fibroblast growth factor, HCl: hydrochloric acid, VSG: vertical sleeve gastrectomy, RYGB: Roux-en-Y gastric bypass, GIP: gastric inhibitory polypeptide, GLP-1: glucagon-like peptide 1, GLP-2: glucagon-like peptide 2, OXM: oxyntomodulin, PYY: peptide YY, ↑: increase, ↓: decrease, (-): no change, ?: unknown.

## Data Availability

Not applicable.
